# The impact of helminths on the response to immunization and on the incidence of infection and disease in childhood in Uganda: design of a randomized, double-blind, placebo-controlled, factorial trial of deworming interventions delivered in pregnancy and early childhood [ISRCTN32849447]

**DOI:** 10.1177/1740774506075248

**Published:** 2007

**Authors:** Alison M Elliott, Moses Kizza, Maria A Quigley, Juliet Ndibazza, Margaret Nampijja, Lawrence Muhangi, Linda Morison, Proscovia B Namujju, Moses Muwanga, Narcis Kabatereine, James AG Whitworth

**Affiliations:** ^a^Uganda Virus Research Institute, Entebbe, Uganda, ^b^London School of Hygiene & Tropical Medicine, London, UK,^c^National Perinatal Epidemiology Unit, Oxford University, Headington, Oxford, UK, ^d^Entebbe Hospital, Entebbe, Uganda, ^e^Vector Control Division, Ministry of Health, Kampala, Uganda

## Abstract

**Background:**

Helminths have profound effects on the immune response, allowing long-term survival of parasites with minimal damage to the host. Some of these effects "spill-over", altering responses to non-helminth antigens or allergens. It is suggested that this may lead to impaired responses to immunizations and infections, while conferring benefits against inflammatory responses in allergic and autoimmune disease. These effects might develop in utero, through exposure to maternal helminth infections, or through direct exposure in later life.

**Purpose:**

To determine the effects of helminths and their treatment in pregnancy and in young children on immunological and disease outcomes in childhood.

**Methods:**

The trial has three randomized, double-blind, placebo-controlled interventions at two times, in two people: a pregnant woman and her child. Pregnant women are randomized to albendazole or placebo and praziquantel or placebo. At age 15 months their children are randomized to three-monthly albendazole or placebo, to continue to age five years. The proposed designation for this sequence of interventions is a 2 X 2(x2) factorial design.

Children are immunized with BCG and against polio, Diphtheria, tetanus, Pertussis, Haemophilus, hepatitis B and measles. Primary immunological outcomes are responses to BCG antigens and tetanus toxoid in whole blood cytokine assays and antibody assays at one, three and five years of age. Primary disease outcomes are incidence of malaria, pneumonia, diarrhoea, tuberculosis, measles, vertical HIV transmission, and atopic disease episodes, measured at clinic visits and twice-monthly home visits. Effects on anaemia, growth and intellectual development are also assessed.

**Conclusion:**

This trial, with a novel design comprising related interventions in pregnant women and their offspring, is the first to examine effects of helminths and their treatment in pregnancy and early childhood on immunological, infectious disease and allergic disease outcomes. The results will enhance understanding of both detrimental and beneficial effects of helminth infection and inform policy. Clinical Trials 2007; 4: 42–57. http://ctj.sagepub.com

## Introduction

Worldwide, more than 2 billion people have helminth infections [1,2]. Concern regarding effects on anaemia, nutrition, growth and intellectual development, and specific disease syndromes, has led to increasing advocacy for mass deworming [3], with new recommendations for treatment in two special, relatively unstudied groups - pregnant women and preschool children [4]. Our study addresses the effects of treatment in these two groups.

Most people with helminths are unaware of their infection, probably because helminth-induced immunomodulation reduces host responses, allowing parasite survival and minimizing tissue damage [5]. This immunomodulation may “spill-over”, altering responses to unrelated organisms (viruses [6], bacteria [7] and mycobacteria [8–10]) and vaccines [11–15]).

We aim to determine whether these immunomod-ulating effects are sufficient to reduce the efficacy of immunization and increase susceptibility to infectious diseases in childhood; and, if so, whether they can be removed by deworming. In particular, it is proposed that the high prevalence of helminth infections in the tropics contributes to the low efficacy of Bacille Calmette-Guérin (BCG) immunization and high incidence of tuberculosis in this region [16–18]. BCG is frequently given to neonates, before acquisition of helminth infection, so the finding that exposure to helminths *in utero* can influence the neonatal response to BCG may be particularly important [19,20], but the implications of observed changes in immune responses for vaccine efficacy are not known. We planned, therefore, to study both the effects of maternal helminths and of deworming during pregnancy, and the effects of acquisition of helminths and of deworming in early childhood, on responses to immunization and on disease incidence in childhood.

Helminth infections are generally considered detrimental. However, recent evidence suggests that they may have some benefits, protecting against abnormal immune responses associated with atopic and autoimmune disease [21–23]. Indeed, trials to investigate the treatment of such conditions with helminths are in progress [24,25]. Helminths may also have a beneficial effect on the response to other pathogens, perhaps protecting against severe malaria [26], or modulating the progression of HIV disease [27].

This trial is therefore designed to investigate the balance of detrimental and beneficial effects of early exposure to helminths and of their treatment. Here we describe how the design of the trial evolved in response to changes in policy regarding deworming in pregnancy, to preliminary findings, and to results from concurrent research. We show that the final protocol involves a novel factorial design, examining related interventions not in the same study subjects, but in pregnant women and their offspring. This design gives efficiency with regard to logistics, cost and trial burden in the community, but is also pertinent to policy, as interactions between the interventions, or additive effects would be important during implementation.

## Hypotheses

Our initial hypothesis anticipated that maternal and childhood helminth infection would have detrimental effects on the response to childhood immunizations and to infectious diseases in infancy. However, our preliminary findings [23], and results from concurrent research [21,22,26,27], suggested possible benefits in relation to disease mediated by poorly regulated inflammatory responses. Thus our current hypotheses, amended in response to preliminary findings, are that 1) maternal and childhood helminth infections reduce the effectiveness of childhood immunizations and increase susceptibility to viral and bacterial infectious diseases, while reducing the incidence of diseases mediated by poorly-regulated inflammatory responses; 2) treatment of maternal and childhood helminth infection improves the effectiveness of childhood immunizations and modulates disease incidence in childhood, with both beneficial and detrimental effects.

## Design

The study was designed in 2000/2001 to evaluate presumptive treatment with albendazole in pregnancy and early childhood and commenced in June 2002. Results from 104 women enrolled using this design (the "preliminary study") have been reported [20,23]. In September 2002 the World Health Organization (WHO) recommended that pregnant and lactating women with schistosomiasis no longer be excluded from treatment, but be given praziquantel individually or during mass treatment [4]. Therefore, the study was discontinued and redesigned to include the use of praziquantel in pregnancy. The revised design is presented here. Recruitment using the revised design (the “main study”) ran from April 2003 to November 2005.

**Figure 1:**
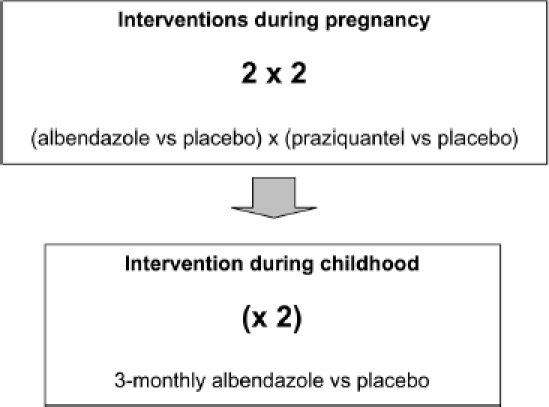
A clinical trial with three randomised, placebo-controlled treatments at two times: a 2 X 2(X2) factorial design

The study is a clinical trial with three randomised interventions at two times, two in pregnant women, and one in their children. For this we propose the designation, a 2 X 2(x2) factorial design (Figure 1).

1. **During pregnancy**, women are randomized toalbendazole versus placebo and praziquantelversus placebo: women receive a) praziquantel + albendazole, b) praziquantel + placebo, c)albendazole + placebo, or d) placebos only. Allwomen are treated with both drugs after delivery.
2. **At age 15 months** their children are randomized to three-monthly albendazole or placebo, tocontinue to age five years. This randomization isindependent of the mother's. Children providestool samples at annual visits and are treated forhelminths found.

The two intervention times create two phases. The first addresses effects of maternal helminths and maternal treatment, with immunological outcomes measured at age one year, and clinical events analysed from age zero to 15 months. The second addresses the duration of effects of maternal helminths and maternal treatment, and effects of acquisition of helminths and of three-monthly treatment with albendazole in childhood.

## Setting

**Figure 2:**
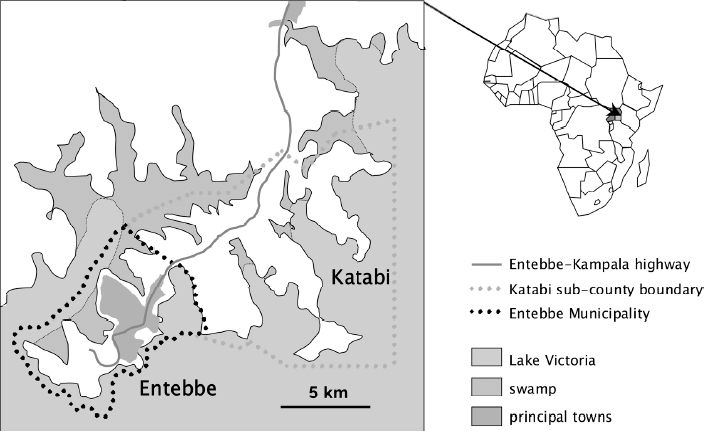
Study setting

The study area comprises Entebbe Municipality and Katabi subcounty, a peninsula in Lake Victoria, Uganda (Figure 2) occupied by semiurban, rural and fishing communities. The prevalence of helminths among pregnant women is 66% [23] (Schistosoma mansoni, 23%; hookworm, 38%; Mansonella perstans, 22% [20]); the prevalence of HIV, 13% [28]. Malaria, pneumonia and diarrhoea are common in young children. Atopic disease is expected to be sufficiently common to be assessed as an outcome [23].

## Recruitment and follow-up

Participation of the Ministry of Health, Entebbe Hospital and the community were, and are crucial to preparation for the study, recruitment and follow-up. Before the study began, the research team collaborated with the Ministry and Hospital to provide training in prevention of mother-to-child HIV transmission (PMTCT) and implement PMTCT using nevirapine [29] in Entebbe. Concurrently, maternity staff participated in planning the study and trained in data and sample collection. Research procedures were integrated into antenatal and maternity routines.

Meetings were also held with community leaders. The study area comprises 57 villages, each with an elected executive. Each committee appointed volunteer assistants, two at first, supplemented as the study expanded, to a total of approximately 150 volunteers. Volunteers visit participants twice a month to make simple checks on the baby's health, and attend monthly meetings to provide feedback. They receive a bicycle and a small allowance. Their work is supervised by full-time field-workers, and verified by routine checks with participants and spot-checks at their homes.

**Figure 3:**
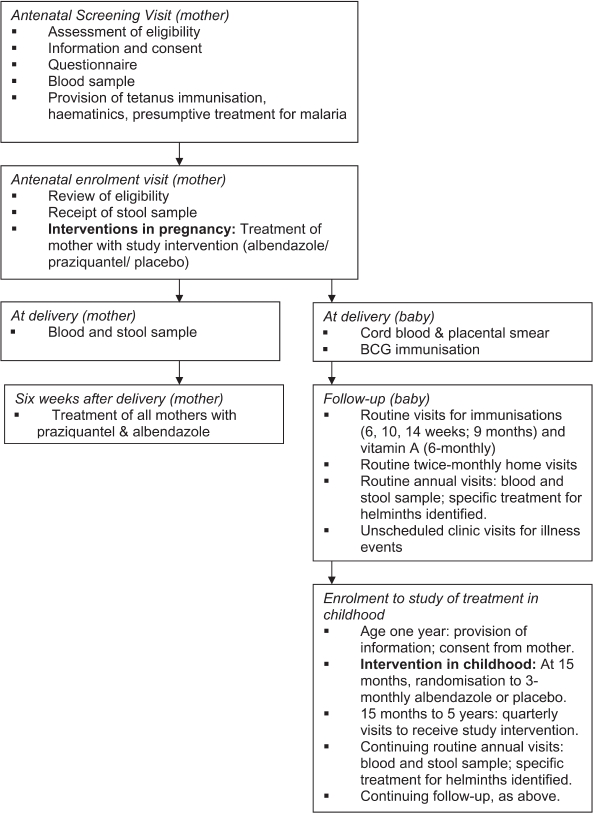
Study procedures

Study procedures are outlined in Figure 3. Routine follow-up visits are at six, 10 and 14 weeks (polio, *Diphtheria*, tetanus, *Pertussis, Haemophilus* and hepatitis B immunization), six months, nine months (measles immunization) and one year. Thereafter children are seen quarterly, to receive study medication; vitamin A is given six-monthly. Mothers bring participating children for interim visits if they are sick.

Nevirapine for PMTCT and antiretroviral therapy, if required, for HIV-positive participants, are provided through the Hospital.

## Information and consent

Written and verbal information is provided in English and the vernacular. Documents written in the vernacular are checked by back-translation. Consent is recorded by signature or thumb-print. Before enrolment, women consent for participation during pregnancy and for their expected infants' participation to age one year. There is no lower age limit for inclusion: all pregnant women (including those younger than 18 years) are entitled to give consent in Uganda, and investigation of the interventions among a representative population, including the youngest women whose helminth prevalence might be highest [30], was considered appropriate. After the baby is delivered, mothers are given written information to take home for the fathers. When infants are one-year old, information is provided, and consent obtained, for the trial of treatment in childhood. The mother or father (or guardian if both are deceased or unavailable) consents for the child.

## Parasitology

Stool and blood samples are obtained from women at enrolment and delivery; from children at annual visits. Stools are examined using the Kato-Katz method [31,32] and charcoal culture for *Strongyloides* [31,33]. Two Kato-Katz slides are prepared from each sample, each examined within 30 minutes for hookworm, the following day for other parasites. Blood is examined for *Mansonella* by a modified Knott's method [34]. Intensity of infection is assessed by egg counts in stool and microfi-larial counts in blood.

## Interventions

### Deworming women during pregnancy and after delivery

The interventions during pregnancy are given as a single treatment, at any time during the second or third trimester.

The randomization code was prepared by the trial statistician using Stata version 7 (College Station, Texas, USA). Numbers were allocated in blocks of 100 to four groups:

1. Praziquantel + albendazole
2. Praziquantel + placebo matching albendazole
3. Placebo matching praziquantel + albendazole
4. Placebo matching praziquantel + placebomatching albendazole

Chewable albendazole and matching placebo tablets were supplied in bulk (GlaxoSmithKline, Brentford, UK). Praziquantel tablets, (Medochemie Ltd, Limassol, Cyprus) were used to prepare identical praziquantel 300 mg and placebo capsules (Almedica Europe Ltd, Deeside, UK). Colleagues in Entebbe, not otherwise involved in the study, prepare opaque, sealed envelopes, numbered according to the randomization code, containing a single dose of albendazole 400 mg (or matching placebo) and 12 capsules of praziquantel (or matching placebo).

Interviewer-counsellors allocate treatment in order of the randomization sequence to women enrolling at the antenatal clinic and observe the treatment. Each woman receives 400 mg albendazole (or matching placebo) and praziquantel capsules (or matching placebo) equivalent to a dose of 40 mg/kg; leftover capsules are discarded.

Six weeks after delivery all women receive albendazole 400 mg and praziquantel 40 mg/kg, with additional anthelmintic treatment if indicated by stool results (such as prolonged treatment for *Strongyloides)*. This was considered necessary, to avoid prolonged delay in treatment of women for potentially damaging helminth infections. Of concern for interpretation of the results of the trial is the possibility that there might be an effect on the infant, mediated through breastfeeding, of treating the mother after delivery. By treating all women after delivery, any such effects are kept as similar as possible between the intervention groups (although, unavoidably, the reduction in worm loads in those treated during pregnancy may mean that effects of the second treatment are not identical between groups). A second option, treating only those not treated during pregnancy, would have meant that any effect of treatment during breastfeeding would be confined to the groups not treated during pregnancy, creating uncertainty about whether treatment during pregnancy or after delivery was more important in determining outcomes in the children. The third option, not treating women after delivery, or waiting for, say, a year, was not considered to be ethical, due to the resulting neglect of treatment or prolonged delay.

## Deworming children aged 15 months to five years

The intervention in childhood is albendazole or placebo given three-monthly.

A second randomization code, for treatment of children, was prepared by the trial statistician using Stata version 7. Study numbers were allocated in blocks of 80 to two groups: albendazole or placebo.

Syrups for children under two years are supplied by GlaxoSmithKline, ready-labelled with the randomization code. Three bottles (15, 18 and 21-month doses) are provided for each child. Each contains 10 mL syrup, equivalent to 400 mg albendazole.

Chewable tablets of albendazole or matching placebo are supplied in bulk for older children. The second randomization code is used by colleagues in Entebbe, not otherwise involved in the study, to prepare opaque, sealed envelopes, numbered according to the code, containing albendazole tablets (or matching placebo) to be taken three-monthly by children aged two years and above.

Children attending at 15 months are allocated treatment by the nurse in charge in order of the second randomization sequence. Children receive 200 mg (5mL) albendazole syrup (or matching placebo) at age 15, 18 and 21 months; the remaining syrup from each bottle is discarded. Children receive 400 mg albendazole or matching placebo three-monthly from age two to five years. Nurses give and observe the treatments.

Stool samples from children are examined at each annual visit; helminths found are treated.

All clinical staff and participants will remain blinded to treatment allocations for women and children until the study is complete.

## Outcomes

The principal outcomes are:

1. Immunological responses to BCG and tetanusimmunization
2. Incidence of infection in childhood with malariaand *Mycobacterium tuberculosis*.
3. Incidence of infectious and atopic disease eventsin childhood (pneumonia, diarrhoea, malaria,measles, tuberculosis and vertical HIV transmission; atopic eczema, urticaria, allergic rhinitisand conjunctivitis, wheeze).

Secondary outcomes are anaemia, growth and development.

### Immunological responses to childhood immunisations

Cellular responses to antigens from BCG and to tetanus toxoid will be measured using whole blood cytokine assays [20,35], providing data on the type and quantity of cytokine produced. Measurements will be made in samples obtained at age one, three and five years.

BCG is a live, attenuated strain of *Mycobacterium bovis*. Immunization usually leads to scarring at the inoculation site and sometimes to complications such as abscess formation, lymphadenopathy or disseminated disease [36]. Examining our preliminary data, we realized that helminth-induced changes in the response to BCG might influence not only cytokine responses, but also scar size [20] and perhaps the incidence of complications. Therefore, in the final protocol, these outcomes are systematically documented at age six weeks and one year, and as illness events if appropriate.

Tetanus immunization induces antibody production, required for its beneficial effect. Antibody will be measured at age one, three and five years.

Assessment of responses to measles immunization will be performed in a substudy.

### Incidence of infection

Helminth infection may influence disease expression for malaria [26], and our preliminary data suggest a possible beneficial effect on disease incidence in children aged two to three years (unpublished data). However an effect on incidence of infection is perhaps unlikely, since this depends on biting by infected mosquitoes. To make this distinction, malaria parasitaemia will be determined at annual visits. In view of the potential public health importance of understanding the effects of helminths on malaria, measurement of anti-malarial antibody was added to the planned measurements, in the final protocol, as an additional surrogate measure of incidence of infection [37].

On the other hand, recent data suggest that BCG immunization may protect against infection with *Mycobacterium tuberculosis*, as well as against disease [38]. If so, helminth infection may modulate this protective effect. Thus, in addition to the outcome of tuberculosis disease (planned from the outset, and discussed below) we will estimate the incidence of tuberculosis infection at annual visits by examining the cellular response to antigens (such as early secreted antigen-6) present in *Mycobacterium tuberculosis* but not BCG.

### Incidence of disease events

The initial protocol focussed on infectious disease outcomes. In response to preliminary results and concurrent research, atopic diseases outcomes were added.

Disease events are documented at clinic visits *Pneumonia* is defined as cough, with difficulty in breathing, and fast breathing (defined by age), with or without abnormal breath sounds [39]. X-rays distinguish clinical and radiological pneumonia [40] *Diarrhoea* is defined by the mother's report, with stool frequency recorded [41] *Malaria* is defined as fever with parasitaemia, but this definition will be reviewed as data on asymptomatic parasitaemia at routine visits accrues, allowing a definition related to asymptomatic parasite counts to be developed [42] *Measles* is defined by standard clinical criteria, confirmed by measurement of specific IgM [43] *Tuberculosis* suspects are investigated using gastric aspirates, lymph node aspirates, or material from other sites, for culture using BACTEC™ (Becton Dickinson, Sparks, Maryland, USA). Where positive cultures cannot be obtained, a diagnostic algorithm is used [44,45].

Data from clinic visits are supported by data collected at twice-monthly, community visits.

Vertical transmission of HIV infection is determined by viral RNA assays (HIV-1 RNA 3.0 assay; Bayer plc, Newbury, UK) in cord blood and at age six weeks; by HIV-antibody tests at age 18 months.

Atopic eczema, urticaria, allergic rhinitis, allergic conjunctivitis and wheeze are defined using criteria from the International Study of Asthma and Allergies in Childhood (ISAAC) [46], modified to suit our young age-group. Documentation of clinic events will be complemented by ISAAC-based questionnaires at age one and four years.

### Anaemia, growth and development

Our principal objectives relate to immunological, infectious and atopic disease outcomes. However, effects on anaemia, growth and development are important for evaluation of overall risks and benefits of deworming.

Haemoglobin is measured in women at enrolment and delivery; in cord blood, and in children at annual visits.

Growth is assessed by birthweight, weights at all routine visits, and measurements of height, head circumference and mid upper-arm circumference at annual visits.

Intellectual function is assessed at age 15 months, by trained clinical staff, using a culturally appropriate tool (the Kilifi Developmental Inventory; Wellcome Trust Unit, Kilifi, Kenya). A new tool will be developed for assessment in children aged five years.

## Statistical considerations

The analyses will have two components: analysis of the intervention trial (which is randomized) and analysis of the effects of helminths (which is observational).

Major analyses will be conducted for each phase of the study.

1. When all children complete age 15 months, analyses will address effects of maternal helminths and maternal treatment on outcomes in infancy.
2. When all children complete five years, analyses will address the duration of effects originating in pregnancy, and effects of acquisition of helminths and of three-monthly albendazole between age one and five years.

Unblinded analyses conducted before the end of the trial, such as Phase 1 analyses, will be performed by the trial statistician. Other staff will see only aggregated data.

### Analysis of the interventions in pregnancy and of maternal helminth infection

The interventions in pregnancy will be analysed by intention to treat. Effects of presumptive treatment on study outcomes for the whole study population will be examined. Three subgroup analyses are planned, as follows, because effects are expected to be stronger when mothers have susceptible species:

1. effects of albendazole if mother had any speciessusceptible to albendazole at enrolment;
2. effects of albendazole if mother had hookwormat enrolment (the commonest infection, mostresponsive to albendazole [20]);
3. effects of praziquantel if mother had schistoso-miasis at enrolment.

Interactions between the treatments will be examined.

It is possible that effects of helminths during pregnancy, established before the interventions are given, may not be altered or reversed by the interventions. Further, some species of helminth, such as *Mansonella*, are unlikely to be affected by the interventions. Thus it will be of interest to examine the effects of helminth infection at enrolment on the study outcomes, in addition to the effects of the interventions on the study outcomes. The analysis of the effects of helminths at enrolment will observational. Differences in effect according to helminth species and intensity of infection, will be examined. This analysis will use logistic or Poisson regression or Cox proportional hazards methods (as appropriate to each outcome), to take account of potential confounding factors including maternal age and tribe, maternal malaria or HIV infection during pregnancy, the socioeconomic status of the family and location of residence.

This general approach will be used for analysis of effects of maternal helminths and maternal treatment on study outcomes when children reach 15 months, and again at five years. At five years, analyses will adjust for the acquisition and treatment of helminths in childhood.

### Analysis of the intervention in childhood and of the acquisition of helminths between age one and five years

The intervention in childhood will be analysed by intention to treat, for the whole study population. No subgroup analyses are planned.

Analysis of the effects of acquisition of helminths will employ categories such as never infected versus ever infected; age at first infection; total number of annual visits at which infection is found and will take account of potential confounding factors, as discussed above in relation to effects of maternal helminths.

The effects of the intervention in childhood, and of acquisition of helminths, will be adjusted for effects of the interventions and of helminth status in pregnancy.

Interactions between maternal and childhood interventions will be examined.

### Twin and triplet pregnancies

Women with twin or triplet pregnancies are enrolled, and treatment given as for singleton pregnancies. In the analysis allowance will be made for "clustering" by mother.

### Sample-size

Considerations regarding sample size evolved with the change in study design to include praziquantel as a second intervention in pregnancy, and with the acquisition of data from the preliminary part of the study, as outlined below.

A cohort of 2500 women has been recruited. This sample-size was planned for the initial design (presumptive treatment with albendazole in pregnancy and early childhood), as sufficient to detect effects on immunological outcomes and common disease events, and with the hope of detecting an effect on incidence of tuberculosis in children under five years old. No information was available for incidence of tuberculosis in children in the study area. In a well-documented community in South Africa the incidence in under-fives was 3588/100 000 p.a.; 3.5 times the incidence in adults [47]. The estimated overall incidence in Uganda was 320/100 000 p.a. [48], so a figure of 500/100 000 p.a. for under-fives might be conservative. It was estimated that, for the effect of albendazole in pregnancy, or for a binary, combined variable for exposure to helminths *in utero* and/or in childhood (expected to be about 50%), a study with 1170 children in each arm and median follow-up of three years would have 80% power with *a =* 0.05 to detect a difference in incidence of 250/100 000 to 750/100 000 cases p.a. between groups. This estimate of effect was large. However, the study seeks to examine the possibility that helminths are crucial to the large difference observed in efficacy of BCG between temperate regions (often above 70%) and the rural tropics (close to 0%) [61]. If this was so, then a large effect was expected. It was noted that, for comparison of the effects of three-monthly albendazole versus placebo in childhood, extension of the follow-up period might be required.

Following the change in study design and acquisition of data from the preliminary study the power of the study to detect important effects and interactions between interventions was reviewed, assuming follow-up to age five years. In the preliminary study, approximately 90% of enrolled mothers were seen at delivery and 85% had live babies who entered follow-up. Of live babies, 75% were seen at one year. Although follow-up in the main study has been better than in the preliminary study, the figures from the preliminary study (with an estimate of 10% loss to follow up per year after one year) were used when the power of the study was reviewed. Estimates may, therefore, be conservative.

Preliminary results suggest that the sample-size will be adequate for immunological objectives. Following neonatal BCG immunization, we observed a difference in mean log_10_ production of the cytokine, gamma interferon, in response to mycobacterial antigens, of 0.64 log_10_ comparing infants of mothers with hookworm to infants of mothers without worms, and a difference of 0.28 log_10_ between infants of mothers with hookworm who did, or did not receive albendazole [20]. Estimating attendance of 1594 infants at one year, and 1046 children at five years, and standard deviations of 0.8 log_10_ for mean log_10_ cytokine responses, we anticipate 80% power with *a =* 0.05 to detect differences between intervention arms at one year (and five years) of 0.11 (0.14) log_10_ cytokine response for the whole population, 0.16 (0.19) log_10_ for the subgroup whose mothers had hookworm and 0.26 (0.33) log_10_ for those whose mothers had schistosomiasis.

For clinical outcomes, Tables 1 and 2 illustrate the smallest treatment effects that the study will detect, with 80% power and *a =* 0.05. The figures apply to effects of either maternal treatment, and, where given for all children aged one to five, to the intervention in childhood. The study has power to detect small effects, except for tuberculosis, where the effect size would have to be large; and to detect interactions between treatments for most variables: for example, an interaction effect of 1.59 for eczema [49].

**Table 1:** The incidence of infectious and atopic diseases in childhood. Estimates of intervention effect sizes that can be detected in the cohort

	Infants to age one year							Children aged one to five years						
		All infants		Infants of mothers with hookworm		Infants of mothers with *S. mansoni*			All children		Children of mothers with hookworm		Children of mothers with *S. mansoni*	
	Expected rate, placebo group per year	Expected pyr	RR	Expected pyr	RR	Expected pyr	RR	Expected rate, placebo group per year	Expected pyr	RR	Expected pyr	RR	Expected pyr	RR
Pneumonia	0.25	1860	0.76	874	0.66	354	0.49	0.1	7948	0.81	3736	0.73	1510	0.59
Diarrhoea	1.9		0.91		0.87		0.80	1		0.94		0.91		0.86
Malaria	0.5		0.82/1.19		0.75/1.29		0.62/1.47	0.8		1.07		1.11		1.17
Eczema	0.25		1.28		0.70		2.19	0.05		1.30		1.76		2.31
Wheeze	0.08		1.51		2.20		3.14	0.04		1.34		1.76		2.31
TB infection	0.03		0.38		0.17		Not possible	0.03		0.67		0.54		0.32
Tuberculosis	0.005		Not possible		Not possible		Not possible	0.005		0.29		0.06		Not possible

The table shows expected disease incidence rate in the placebo group, and expected total (intervention + control) person years of follow-up for infants and children, for the whole study population and for subgroups whose mothers had hookworm or schistosomiasis. The smallest effects that the study has 80% power to detect, with *a =* 0.05 are shown as rate ratios. The direction of effect shown is that predicted by our hypothesis; both directions of effect are shown for malaria in infancy where either would be plausible. TB: *Mycobacterium tuberculosis*. Not possible: insufficient person years available in the study to detect an effect with 80% power and *a =* 0.05.

**Table 2:** Anaemia, growth and development. Estimates of intervention effect sizes that can be detected in the cohort

		Infants aged one year (15 months for development scores)							Children aged one to five years						
		All infants		Infants of mothers with hookworm		Infants of mothers with *S. mansoni*			All children		Children of mothers with hookworm		Children of mothers with *S. mansoni*	
	Expected mean (SD) value in placebo group	Expected number	Difference	Expected number	Difference	Expected number	Difference	Expected rate, placebo group per year	Expected number	Difference	Expected number	Difference	Expected pyr	Difference
Haemoglobin (g/dl)	10.3 (1.38)	1594	+0.19	748	+0.28	286	+0.46	11 (1.8)	1046	+0.31	552	+0.43	188	+0.74
Weight (kg)	9.4 (1.36)		+0.19		+0.28		+0.45	15.4 (1.7)		+0.29		+0.41		+0.69
Development (psychomotor)	60 (7)		+0.98		+2.43		+2.32							
Development (language)	220 (185)		+26.0		+37.9		+61.3							

The table shows expected mean values in the placebo group, and numbers of infants and children (intervention + control) expected at one and five years, for the whole study population and for subgroups whose mothers had hookworm or schistosomiasis. The smallest increases in parameters that the study has 80% power to detect, with a = 0.05 are shown. For developmental indices, tools for five-year olds have not yet been developed.

Effects of helminths are expected to be stronger than effects of the interventions, so, for common outcomes, the anticipated sample-size will allow for inclusion of confounding factors in the analysis.

On the basis of these considerations, the trial steering committee decided to retain the planned sample-size.

## Ethical considerations

Given uncertainty regarding the immunological benefits and risks of treating helminths in pregnancy and early childhood, a placebo-controlled trial was undertaken. There was concern that interventions in pregnancy might lead to serious adverse birth outcomes, so a data monitoring committee (DMC) was established to monitor these and other serious adverse events. Further, because a transient increase in HIV load following anthelmintic treatment [50] might cause an increase in vertical HIV transmission, results for HIV RNA assays performed in HIV-exposed infants at six weeks of age are also reported to the DMC. Ethical considerations specific to the interventions, are as follows.

### Albendazole versus placebo in pregnancy

Albendazole treatment during pregnancy can have a benefit for anaemia in areas of high hookworm prevalence [51]. Therefore women with haemoglobin below 8 g/dL are excluded and referred for treatment of hookworm (Table 3); haematinics and presumptive treatment for malaria (interventions with a similar or greater benefit for anaemia in pregnancy [51,52]) are provided.

**Table 3:** Inclusion and exclusion criteria

Inclusion criteria	Exclusion criteria
Resident in study area	Anaemic: haemoglobin <8 g/dl
Planning to deliver in Extebbe General Hospital	Clinically apparent severe liver disease
Willing to participate in the study	Diarrhoea with blood in the stool
Willing to know her HIV status	Midwives assess pregnancy to be abnormal
In the second or third trimester of pregnancy	History of adverse reaction to anthelmintic drugs
	Already enrolled in an earlier pregnancy

Additional suggested benefits of albendazole in pregnancy include improvements in birthweight and infant survival [53–55] but these have not been demonstrated in controlled trials.

Teratogenicity is a concern in the use of benzim-idazoles during pregnancy (reported in animals, but not in humans) [56]. Therefore women are enrolled after the first trimester.

### Praziquantel versus placebo in pregnancy

Before our study started, no investigations of praziquantel treatment in pregnancy had been performed. Although presumed safe, based on animal studies, its use was therefore avoided in pregnancy; breastfeeding was discontinued for 72 hours after treatment to avoid toxicity to the infant. The WHO consultation of 2002 recommended use of praziquantel during pregnancy based on a lack of evidence of toxicity in a small number of pregnant women treated for cysticercosis, and in large numbers treated inadvertently during mass-treatment campaigns, and on concerns regarding prolonged neglect of treatment of schistosomiasis in women of child-bearing years [4]. However, the anecdotal data for cysticercosis do not provide insight into the effects of killing intravenous schis-tosomes. In particular, the systemic and transpla-cental immunological effects of treatment of schistosomiasis in pregnancy are unknown; the benefits and risks for maternal and neonatal health, remain uncertain.

Persistent diarrhoea with blood is the pathological effect of chronic schistosomiasis mansoni most likely to show an immediate response to treatment, so women with these symptoms are excluded and referred for investigation and treatment (Table 3).

Prolonged neglect of treatment is avoided in this study by treatment of all women after delivery.

### Three-monthly albendazole versus placebo in children aged 15 months to five years

Since our study was planned, results have been published describing a trial of deworming in children under five years old in Zanzibar [57,58]. Outcomes included growth, anaemia and cognitive development. This study showed no statistically significant, overall benefit of deworming. Subgroup analyses found that mebendazole was associated with a reduction in wasting malnutrition in children under 30 months, and in moderate anaemia for children under 24 months. The observation of benefit principally in the youngest children, with the lightest worm burdens, was the reverse of the result expected. The authors suggest that the youngest children might be particularly vulnerable to the effects of helminths, or that inflammatory processes during the response to initial helminth infections might have a particularly severe effect. The inconclusive and unexpected nature of these results, added to the possibility that intensive deworming may have adverse effects on atopic disease and on severity of malaria, suggests equipoise of potential beneficial and detrimental effects, supporting the need for further study.

### Ethical approval

Approval was given by the Science and Ethics Committee, Uganda Virus Research Institute; the Uganda National Council for Science & Technology; the London School of Hygiene & Tropical Medicine.

## Discussion

There is increasing advocacy for deworming, with a recent focus on pregnancy and early childhood. Together with concern regarding effects on anaemia, nutrition, growth and development, there is intense interest in the hypothesis that helminths impair the response to vaccines and increase susceptibility to infectious diseases, while perhaps protecting against immunologically-mediated conditions such as atopic and autoimmune disease and severe malaria. There is increasing evidence of the importance of prenatal exposures (including exposure to helminth infection) for health outcomes in later life. Despite the potential importance of such effects for health policy, few investigations have been conducted to determine the magnitude of the proposed effects of helminths, or the ability of treatment to modify them.

Our study design has three aspects of particular note. First, is the intention to examine effects of helminths and of their treatment in the same study. Analysis of the effects of helminths is pertinent, because their magnitude is uncertain, but must take account of confounding with factors such as poverty and malnutrition. Examining the results of treatment in a randomized trial may provide more rigorous evidence of helminth-induced effects. This is illustrated in Table 4 by a detailed breakdown of results regarding the effects of maternal helminth infection on the incidence of infantile eczema from our preliminary study [23]. Although based on small numbers, and given the proviso that the analysis of effects of helminths requires adjustment for confounders, the results seem to show, in the placebo group, the albendazole-treated group and overall, that the rate of eczema was about five times higher in infants whose mothers never had helminths (none-none) compared with those whose mothers had persistent helminths (any-any). In all helminth-status groups, the rate of eczema was higher in the albendazole-treated group than the placebo group (except none-any where both rates were zero and numbers small). A small effect of albendazole among those whose mothers never had helminths (none-none) may be attributed to clearing of infections not detected by the single stool examination; a large effect among those whose mothers cleared their infection (any-none) suggests that the risk of eczema was highest in infants of mothers who had helminths that were cleared by treatment. Thus data suggesting an effect of helminths can be supported by evidence suggesting a reversal of their effect by treatment. At the same time, the effects of a policy of presumptive treatment in pregnancy can be considered.

**Table 4:** Associations between incidence of infantile eczema, maternal helminth infection and treatment with albendazole during pregnancy

			Incidence rates for eczema in infants age 0–15 months* (per 100 person years)					
Maternal helminth infection status			Mother received placebo		Mother received albendazole		Overall	
In pregnancy	At delivery	Person years of follow-up	Number of episodes	Rate (95% CI)	Number of episodes	Rate (95% CI)	Number of episodes	Rate (95% CI)
None	None	19.1	5	53.8 (22.4–129.3)	9	91.4 (47.6–175.7)	14	73.1 (43.3–123.5)
None	Any	2.3	2	0	0	0	0	0
Any	None	4.1	0	0	6	291.4 (130.9–648.7)	6	145.8 (65.5–324.6)
Any	Any	36.8	2	10.4 (2.6–41.7)	3	17.1 (5.5–52.9)	5	13.6 (5.7–32.7)

*Data from the preliminary study. Multiple events occurring in the same infants are included in these crude rates. A more detailed analysis, allowing for multiple episodes using a random effects model, is presented elsewhere [23].

Second, we have chosen to examine effects of both albendazole and praziquantel during pregnancy in the same trial. This is relevant, since deworming policy is likely to recommend both in regions where geohelminths and schistosomiasis are coprevalent [59,60]. However, there is increasing evidence that immunological effects vary between helminth genera. Studying these two treatments in a factorial design will allow us to examine their effects both separately and together.

Third, we have chosen to incorporate a trial of the effects of deworming in young children in the same cohort as a trial of deworming in pregnancy. This again reflects emerging policy. Some losses are inevitable between pregnancy and childhood, so the number of people randomized in the childhood component differs from the number randomized during pregnancy, and the people themselves, being mother and child are different. Despite this, the interventions are related and may have interacting or additive effects; hence the proposed, novel designation, a 2 X 2(x2) factorial design. This approach is, as far as we know, unprecedented, but is particularly relevant to the implementation of related interventions in mothers and children, when important interactions, such as potentiation of effects of interventions in pregnancy by intervention in childhood, may occur.

In addition to achieving scientific goals, the combining of objectives is a matter of expediency. Despite uncertainty regarding the benefits and risks of deworming in pregnancy and young children, enthusiasm for these policies means that the window of opportunity for conducting such studies is likely to be short. Indeed, our ability to detect effects of the acquisition and treatment of helminths in young children may be limited by the on-going provision of albendazole in Uganda by community-based distributors and on twice-yearly "child days"; this extra treatment is, however, monitored in the study, and uptake is likely to be similar between the randomized groups.

The study has several potential limitations. First, the sample-size may be insufficient for evaluation of rare outcomes. Interpretation of the effects of helminths on the response to immunization depends, ultimately, on incidence of disease, thus rare outcomes of particular interest include tuberculosis and measles. The likely incidence of tuberculosis infection and disease in the cohort remains low and uncertain. The incidence of measles may increase in older children as vaccine-induced protection wanes, but depends, among other things, on vaccine coverage in the community. Similarly, while effects on infantile eczema are of interest, effects on long-term risk of asthma will be of greater importance. Follow-up is currently planned to age five years, but longer follow-up may be required to obtain conclusive results for such conditions.

Second, the results may not all be generalisable. Communities differ in important respects, such as species and intensity of helminth infection, nutritional status, and exposure to non-pathogenic mycobacteria that may influence immunity to tuberculosis [61], to pathogens (such as malaria and HIV), to allergens, and to pollutants that may influence responses to allergens [62–64]. Similar studies are needed in other settings.

Advocacy for deworming has created a climate in which delayed or infrequent deworming may be thought unethical. To our knowledge, the only clearly demonstrated benefit of deworming in pregnancy is a reduction in hookworm-associated anaemia and the benefits of frequent de-worming for children under five are uncertain. In the absence of proven benefit, and given the possibility of detrimental effects such as teratogenicity, promotion of atopic disease or increased severity of malaria, placebo-controlled trials remain appropriate. Safeguards to prevent neglect of severe anaemia, or of tissue-damaging infections, can be incorporated into the study design, as described.

This is the first trial undertaken to examine the effects of helminths, and of deworming in pregnancy and young children, on immunological, infectious disease and atopic disease outcomes in children. The design will allow the role of helminths and benefits of deworming to be elucidated in relation to the more conventional outcomes of anaemia, growth and development, also. The results are expected to inform policy and to enhance understanding of both detrimental and beneficial effects of helminth infection.
